# Molecular typing of *Salmonella enterica* serovar Enteritidis isolates from food-producing animals in Japan by multilocus variable-number tandem repeat analysis: evidence of clonal dissemination and replacement

**DOI:** 10.1186/1751-0147-56-31

**Published:** 2014-05-13

**Authors:** Ayumi Kobayashi, Sayaka Takahashi, Masaaki Ono, Kiyoshi Tanaka, Masato Kishima, Masato Akiba, Ikuo Uchida

**Affiliations:** 1 Ishikari Livestock Hygiene Service Center, 3 Hitsujigaoka, Toyohira, Sapporo 062-0045, Hokkaido, Japan; 2 Hokkaido Research Station, National Institute of Animal Health, 4 Hitsujigaoka, Toyohira, Sapporo 062-0045, Hokkaido, Japan; 3 Zen-noh Institute of Animal Health, Ojamachi 7, Sakura, Chiba 285-0043, Japan; 4 National Institute of Animal Health, Tsukuba 305-0856 Ibaraki, Japan; 5 Graduate School of Life and Environmental Sciences, Osaka Prefecture University, 1-58 Rinku-oraikita, Izumisano, Osaka 598-8531, Japan; 6 United Graduate School of Veterinary Sciences, Gifu University, 1-1 Yanagido, Gifu-shi 501-1193, Japan; 7 Disease Investigation Center Subang, Subang 41212, West Jawa, Indonesia

**Keywords:** *Salmonella* Enteritidis, Food-producing animals, MLVA, Clonal replacement

## Abstract

**Background:**

*Salmonella enterica* serovar Enteritidis is a zoonotic pathogen. Human infections are associated with contaminated eggs and egg products. In Japan, since 1989, the incidence of food-borne disease caused by *S.* Enteritidis has increased and a pandemic has occurred; however, little is known about changes that occurred before and after this pandemic event in the dominant lineage of isolates from food-producing animals. This study aimed to determine the *S.* Enteritidis lineages in Japan over the last few decades by using multilocus variable-number tandem repeat analysis (MLVA).

**Findings:**

MLVA was used to analyse 79 *S*. Enteritidis isolates collected from chickens (n = 63), cattle (n = 12), pigs (n = 2), and goats (n = 2) during 1975–2009. The *S.* Enteritidis isolates showed 14 different MLVA allele combinations, which were classified into two major clusters (A and C) and a minor cluster (B). All the 62 isolates in cluster A were isolated after 1988, whereas 13 of the 17 isolates belonging to cluster B and C were isolated before 1989.

**Conclusions:**

The MLVA results showed that cluster C was predominant before 1989, and isolates in cluster A disseminated since 1989 and replaced the previous dominant clone, suggesting that isolates of cluster A originated from imported *S.* Enteritidis infection.

## Findings

*Salmonella enterica* serovar Enteritidis is a zoonotic pathogen that can be transmitted to humans via many different reservoir hosts. Most outbreaks of *S.* Enteritidis infections have been associated with contaminated eggs and egg products [1]. Until the 1980s, *S.* Typhimurium was the serovar most commonly isolated from humans worldwide; however, during the 1980s and 1990s, *S.* Enteritidis emerged as a common cause of salmonellosis, first in European countries and then worldwide [2-4].

Similarly, in Japan, the serotype *S.* Enteritidis has been isolated most frequently, since 1989, from patients with food-borne illnesses [5]. Moreover, *S.* Enteritidis phage type PT8 was the most prevalent human isolate reported until 1988, until PT34 became the predominant phage type; PT1 and PT4 have replaced PT34 as the predominant phage types since 1992 [5]. However, little is known about changes that have occurred over the last few decades in the dominant lineage of isolates from food producing animals in Japan.

Phage typing is a commonly used method for epidemiological surveillance of *S.* Enteritidis infection [6]; however, it requires specialized reagents and laboratory equipment and does not always yield sufficient information for epidemiological purposes. Over the last decade, new techniques in molecular biology have been developed, such as pulsed-field gel electrophoresis (PFGE) [7,8], 2-enzyme ribotyping (*Pst*I-*Sph*I) [9], and multilocus variable-number tandem repeat analysis (MLVA) [10,11]. Of these methods, PFGE is now the gold standard for discriminating among strains at the DNA level [7]. However, based on amplification of a variable number of tandem repeat areas, MLVA is considered to have greater discriminatory power than PFGE and has been proposed as an alternative for genotyping of *S.* Enteritidis [10,11].

In this study, MLVA genotyping was performed to determine the lineages of *S.* Enteritidis before and after pandemics during the 1980s and 1990s. We have provided evidence that clonal dissemination and replacement have occurred among isolates from food-producing animals since 1989.

**Figure 1:**
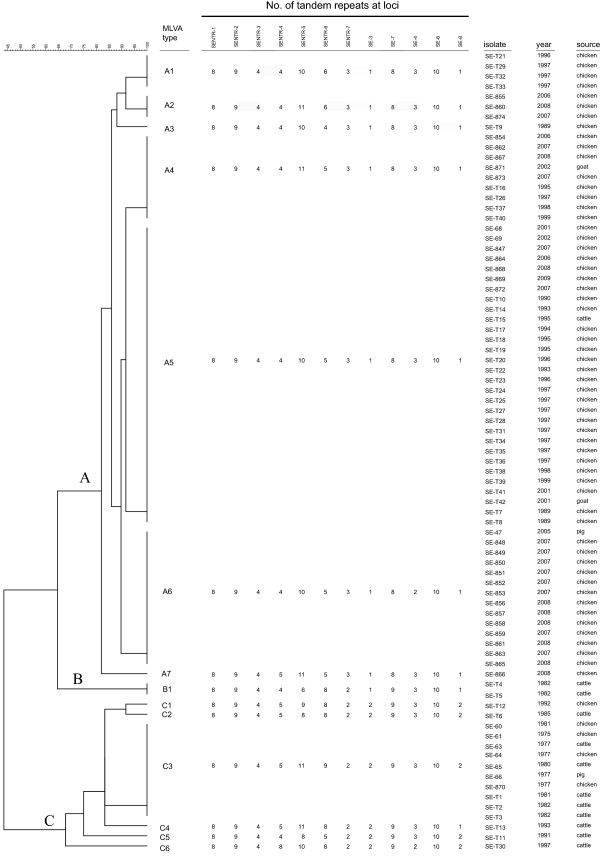
**Dendrogram of 79 *Salmonella *Enteritidis isolates based on MLVA profiles.** The dendrogram is divided into three clusters: **A**, **B**, and **C**. The numbers in the columns SENTR-1 to SE-8 indicate repeat count.

A total of 79 isolates from chickens (63 isolates), cattle (12 isolates), pigs (2 isolates), and goats (2 isolates), which are epidemiologically unrelated, were collected in Japan during 1975 to 2009 in 24 prefectures in Japan (Figure 1), and these strains were used for MLVA. MLVA was conducted by amplification of 12 loci (SENTR-1, SENTR-2, SENTR-3, SENTR-4, SENTR-5, SENTR-6, SENTR-7, SE-3, SE-7, SE-4, SE-6, and SE-8) using previously designed primers [10,11]. PCR products were sequenced to confirm the number of tandem repeats. The number of tandem repeats at each locus was manually determined using Genetyx version 11.0 (Genetyx, Tokyo, Japan), and the motif numbers present in the tandem array were imported into BioNumerics version 6.0 (Applied Maths, Sint-Martens-Latem, Belgium).

The 79 *S.* Enteritidis isolates included 14 different MLVA profiles (Figure 1). Nine distinct MLVA profiles were identified from chicken isolates, 7 were identified from cattle, 2 from goats, and 2 from pigs. A dendrogram was generated using the categorical coefficient and unweighted pair group method with arithmetic means by the BioNumerics software (version 6.0). MLVA profiles were classified into 3 clusters (A, B, and C) delineated with a 67.8% similarity cutoff value (Figure 1). Cluster A consisted of 7 profiles corresponding to 62 isolates, including 58 isolates from chickens, 2 isolates from goats, 1 isolate from cattle, and 1 isolate from pig. Cluster B included 2 isolates from cattle, and cluster C, which was composed of 6 MLVA profiles representing 15 isolates, included 9 isolates from cattle, 5 isolates from chickens, and 1 isolate from pigs.

**Figure 2:**
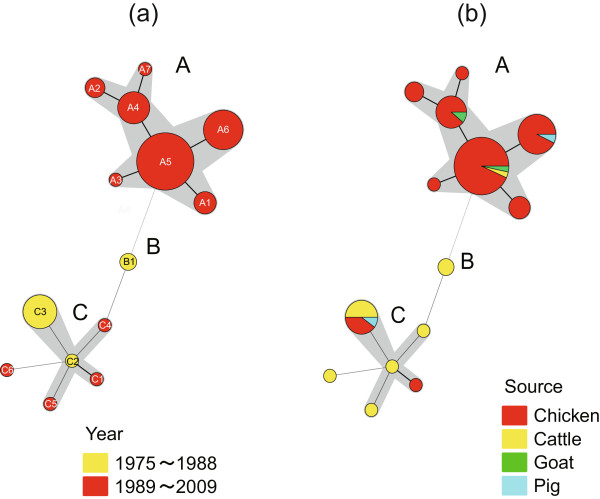
**Minimum spanning tree of 79 *Salmonella *Enteritidis isolates. (a)** Each node represents a unique MLVA profile, indicated by the number in the middle of the circle. The nodal size is proportional to the number of isolates per MLVA profile. The colour within each node reflects the proportion of strains isolated from different periods per node. The length of the branches represents genetic distance (change in loci) between two neighbours. Bold short lines connect two MLVA types differing by a single MLVA locus. Thin longer lines connect double or triple variants, and dotted lines indicate connection between two types differing by four MLVA loci. Clonal complexes were created based on maximum neighbour distance changes at two loci. **(b)** This is the same minimum spanning tree as in Figure 1a. Wedges in circles indicate the proportion of isolates from respective sources with a particular MLVA profile.

A minimum spanning tree (MST) was also created based on the categorical data sets (Figure 2). All the MLVA profiles in cluster A clustered into a single clonal complex in the MST, and all isolates belonging to cluster A were isolated after 1988 (Figure 2). With the exception of MLVA profile C6, profiles in cluster C were clustered into a single clonal complex, and this complex differed from the cluster A complex at 7 loci (Figure 2). The MLVA profile C3 included 5 isolates from cattle, 4 isolates from chickens, and 1 isolate from pigs (Figure 2). All 10 isolates representing profile C3 were isolated before 1983 (Figure 1). Of the isolates in cluster C, 4 isolates that exhibited MLVA profiles C1 (from chicken) and C4, C5, and C6 (from cattle), were isolated in 1992, 1993, 1991, and 1997, respectively (Figure 1). These 4 isolates could have originated from isolates showing profile C3, which was the dominant lineage before 1983 (Figure 1). A profile of cluster B, comprising 2 isolates collected from cattle in 1982, exhibited changes in the neighbour distance at 3 and 4 loci from clusters A and C, respectively (Figure 2). Therefore, the lineages of these 2 isolates from cattle seem to be different from those of clusters A and C, which included isolates from chickens. A proportion of the cluster A isolates isolated after 1988 was statistically different from that of isolates before 1989 (*P* < 0.01, by Fisher’s exact test). Taken together, these results indicate that MLVA cluster C was predominant before 1989 and that clonal dissemination of lineage MLVA cluster A has occurred in food-producing animals in Japan since 1989. Porwollik *et al.*[12] differentiated between two major phage type lineages, including PT4 and PT8, by whole genome microarray analysis. These lineages might correspond to MLVA clusters A and C, respectively. Since PT8 was the most prevalent phage type until 1989 in Japan [5], isolates of this phage type might belong to MLVA cluster C. *S.* Enteritidis phage type PT4 was detected in isolates from chickens imported from England in 1989 [8], and a variety of phage types, including PT4, was observed in isolates from chickens after 1989 [8,13]. Although we have not yet determined the phage type and have not differentiated between imported and native isolates, no isolate classified into cluster A was detected before 1989, suggesting that isolates in cluster A may have originated from imported *S.* Enteritidis infections; consequently, clonal expansion of *S.* Enteritidis might have occurred in chickens and also affected humans.

Since MLVA profiles can be easily stored in a database, routine and long-term epidemiological surveillance with such a method may enable early recognition of potentially epidemic *Salmonella* clones.

## Competing interests

The authors declare that they have no competing interests.

## Authors’ contributions

AK, KT, and IU provided data and discussion for the results and drafted the manuscript. ST, MO, MK, and MA supervised isolation and identification of *S.* Enteritidis. All authors have read and accepted the final manuscript.
